# Bioaccumulation, Biodistribution, Toxicology and Biomonitoring of Organofluorine Compounds in Aquatic Organisms

**DOI:** 10.3390/ijms22126276

**Published:** 2021-06-11

**Authors:** Dario Savoca, Andrea Pace

**Affiliations:** Dipartimento di Scienze e Tecnologie Biologiche, Chimiche e Farmaceutiche (STEBICEF), Università Degli Studi di Palermo, 90100 Palermo, Italy; dario.savoca@unipa.it

**Keywords:** polyfluorinated compounds, perfluorinated compounds, perfluoroalkylic compounds, PFC, ecotoxicity, biomagnification, bioconcentration, environmental impact, pollution, fluorine chemicals

## Abstract

This review is a survey of recent advances in studies concerning the impact of poly- and perfluorinated organic compounds in aquatic organisms. After a brief introduction on poly- and perfluorinated compounds (PFCs) features, an overview of recent monitoring studies is reported illustrating ranges of recorded concentrations in water, sediments, and species. Besides presenting general concepts defining bioaccumulative potential and its indicators, the biodistribution of PFCs is described taking in consideration different tissues/organs of the investigated species as well as differences between studies in the wild or under controlled laboratory conditions. The potential use of species as bioindicators for biomonitoring studies are discussed and data are summarized in a table reporting the number of monitored PFCs and their total concentration as a function of investigated species. Moreover, biomolecular effects on taxonomically different species are illustrated. In the final paragraph, main findings have been summarized and possible solutions to environmental threats posed by PFCs in the aquatic environment are discussed.

## 1. Introduction

The peculiar physicochemical properties of fluorinated organic molecules have opened the way to their large diffusion since the second half of the last century [1]. In particular, organic molecules containing several fluorine atoms along the carbon skeleton, such as poly- and perfluoroalkyl substances (PFAS), have been applied as refrigerants, foam-blowing agents, fire suppressors as well as in fluoropolymeric textiles, paints, and materials for their oil- and water-resistant features [1,2,3]. Together with their industrial diffusion, this family of chemicals, which includes more than 5000 highly fluorinated aliphatic compounds, has found different applications also in consumer products, from cookware and carpets to food-packaging and electronics [2,4].

In this review, we will use the acronym PFCs referring to poly- and perfluorinated compounds where the fluorine atoms are located in different parts of the considered molecule (including alkylic moieties), while the term PFAS will be used to indicate a subclass of PFCs consisting only of poly- and perfluoroalkyl substances.

Unfortunately, the initial underestimation of their environmental impact, together with their large use has caused in the last decades an increased level of environmental contamination from poly- and perfluorinated compounds (PFCs), and in particular from PFAS. In fact, due to the chemical stability of the C-F bond, PFAS are resistant to degradation and represent one of the major classes of persistent organic pollutants (POPs) diffused in the environment [5,6]. However, their environmental behavior, bioaccumulation, and toxicological activity, usually follow different paths compared to other widely studied POPs (e.g., organochlorine and organobromine compounds) [7]. The class of PFAS includes perfluoroalkylic acids (PFAA), with perfluoroalkanoic carboxylic acids (PFCA) and perfluoroalkane sulphonic acids (PFSA) being the most frequently studied compounds [8]. In the new millennium, research activities of laboratories, regulatory authorities, and industry have converged to classify, monitor, and regulate these pollutants to contrast their environmental impact [9].

These activities include the assessment of the qualitative state of the environment, measured by ecological bioindicators to evaluate the stressful influence of PFAS, particularly in aquatic environments. To minimise the impact of PFAS in aquatic ecosystems and along trophic networks, it is essential to verify their presence in various environmental matrices [1,10,11,12,13] and to study their effects on organisms [8,14,15,16,17,18,19].

For the determination of PFAS in aquatic organisms, several studies have been carried out and more sensitive, rapid, and robust extraction and analytical methods have been recently developed [20,21,22,23,24]. Moreover, literature on PFAS environmental impact is continuously foraged by new research on their bioconcentration [25], health effects [26], human exposure, and legal regulation [8]. The purpose of this review is to provide an organized overview of the impact of PFAS, with a specific focus on aquatic biota, evaluated through studies on biomonitoring, determination of contamination and biodistribution profile, evaluation of their bioaccumulative potential, assessment of biomolecular effects caused by these pollutants, and their aquatic half-life. A critical analysis of literature data is illustrated in each relevant section and summarized in the conclusive paragraph highlighting unresolved issues and offering a perspective view for future research.

The fate of PFAS after their release into the environment depends on their transport, partitioning, and transformation processes and its assessment is crucial to define measures to contrast their impact. Due to atmospheric and oceanic transport PFAS have been found in areas far away from the source of contamination [27,28] thus, nowadays, open waters and their sediments are considered relevant pools of these persistent pollutants [3,29,30,31].

The PFAS transport cycle depends on the structure and physicochemical properties of the substance itself as well as on several environmental conditions, including the content of organic carbon, temperature, salinity, and concentration of atmospheric oxidants in the aquatic environment [32,33]. Despite PFAS and their precursors could be subjected to a variety of environmental and biological transformations in different compartments [34,35], they possess a substantial bioaccumulation potential that varies between individual organisms and species and depends both on the mechanisms of active transport of the organisms and on the nature of the different PFAS compounds [36,37].

Assessing the environmental fate of PFAS is crucial to evaluate the risk of exposure, to such persistent pollutants, of animals up to the apex of the trophic network, especially considering that intake often occurs through ingestion. In fact, in addition to exposure linked to contamination of the physical environment (air, water, soil), the direct ingestion of a contaminated food significantly increases the assimilation of the pollutant [38,39,40,41,42,43,44,45,46]. For humans, other routes of PFAS intake are possible through direct exposure to the polluted environment (e.g., inhalation of dust or atmospheric particulate from industrial sources) [38,47,48,49,50,51,52] in addition to the ingestion of contaminated beverages and food, including wild and farmed seafood [1,53,54,55]. For instance, a very recent study based in Tanzania has reported a PFAS contamination level in fish and seafood that would expose humans up to a three-fold amount of perfluorooctanesulfonic acid (PFOS), with respect to the tolerable dose, under the regular fish consumption regimen of 0.016–0.027 kg/capita/day [55]. A similar study, taking into account the contamination of flathead mullet (*Mugil cephalus*), Atlantic mackerel (*Scomber scombrus*), and European plaice (*Pleuronectes Platessa*), hake (*Merluccius merluccius*), and sea bass (*Dicentrarchus labrax*), has reported PFAS contamination levels that were above the tolerable daily intake for toddlers’ diet in Italy [56]. In this context, despite the Organization for Economic Cooperation and Development (OECD) has identified more than 4500 PFAS-related substances [57], only a few of them (<20) are regularly analysed and monitored, thus underestimating the real exposure to PFAS. For example, the percentage of unidentified organofluorine compounds may range from 30 to 90% of the total extracted fluorinated substances in wildlife animals or marine mammals [58,59]. Additionally, it is important to remark that the levels of seafood contamination due to the conservation and transformation processes increase with the market demand of such products [60] thus, considering the diffusion of PFAS in the environment, constant and extensive monitoring of PFAS contamination is required to guarantee food safety to the consumers.

## 2. Solubility, Persistence and Aquatic Half-Life of PFCs

The solubility of PFCs and their bioavailability as freely dissolved substances in either salty or freshwater play an important role in possible deviations between the nominal and measured (under controlled experimental conditions) concentrations of the investigated substance, posing the risk of potential underestimation of the toxic effect PFCs [61]. Indeed, a slow dissolution process could lead to a slow accumulation and metabolization as well as to an increase in the half-life of PFCs (due to the replacement of consumed PFC by newly dissolved PFC). In this context it is important to highlight that, in addition to the high absorption rate of PFCs in organic matter dispersed in water and aquatic species, the ubiquity and persistence of PFCs in water, e.g., about 40 and 90 years for perfluorooctanoic acid (PFOA) and PFOS, respectively, make these molecules difficult to eradicate [62,63]. Inside an aquatic organism, the half-life can depend on the type of metabolism and is more sensitive to the type of functional groups. For instance, the presence of sulfonic or carboxylic acid moieties can increase the half-life of the substance (regardless of the carbon chain length) [63]. The half-life, is also affected by the type of isomer, as seen for PFOA’s and perfluorononanoic acid’s (PFNA) isomers in rainbow trout [64]. In general, the half-life and the rate of elimination from the aquatic organism of the different PFCs vary according to: (i) the type of PFC and the levels of exposure concentration; (ii) the species and sex of the organism; (iii) the type of predominant path of intake in the bloodstream (e.g., aqueous or dietary); (iv) the organs or tissues involved (e.g., gills or intestinal wall); and (v) the experimental design under controlled conditions.

Regarding the type of exposure, a shorter PFOS half-life (11–17 days) was recorded in fish fed with contaminated food compared to half-life recorded after aqueous exposure (29–35 days) [65]. Interestingly, PFAS kinetics were faster in northern leopard frog (*Rana pipiens*) tadpoles, where the half-life ranged from 1.2 to 3.3 days for all investigated chemicals: PFOS, perfluorohexanesulfonic acid (PFHxS), PFOA, and 6:2 fluorotelomer sulfonate (6:2 FTS) [66]. Furthermore, particular attention and should be paid to biotransformation processes since PFCs metabolites could have longer half-lives than their precursors, thus representing new candidates for biomonitoring [13].

The elimination times of PFCs also depend on the size of the organisms and its diet, a factor that is crucial in the biomagnification processes since a chronic dietary intake of PFCs is possible as the trophic level rises. For instance, the half-life of PFOS was 5 months in dolphins [67], 12–15 days in rainbow trout [32], and 29–31 days in marbled flounder (*Pseudopleuronectes yokohamae*) [68]. Similarly, in juvenile rainbow trout, depuration half-lives ranged from 3 to 43 days and increased with the number of perfluorinated carbons present in the chemical, while the bioaccumulation decreased in the following order: PFSAs > PFCAs > perfluoroalkyl phosphonic acid (PFPAs) showing preferential partitioning into blood and liver [69].

Surprisingly, for wild species, the observed half-life of PFCs is longer, probably due to the continuous status of pollution of the environment. For example, the 50% clearance of PFOS from whole fish was greater than 100 days [29,36].

In this context, also the production of chemical alternatives to PFCs does not represent a viable solution to the threats posed by PFCs impact. For instance, the half-life of 6:2 chlorinated polyfluorinated ether sulfonate (F-53B) (a fluorinated compound alternative to PFOS) ranged around 10 days in zebrafish larvae indicating a high persistence potential in aquatic organisms [70].

## 3. Bioaccumulative Potential

**Figure 1:**
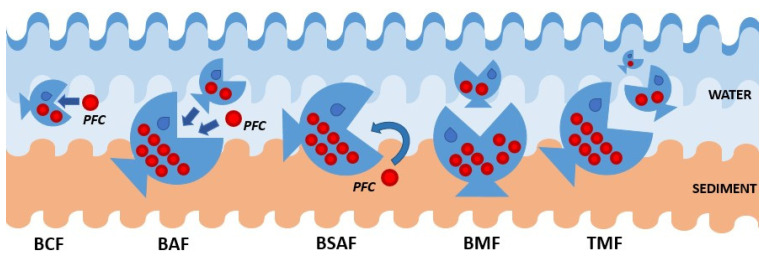
Illustration of bioaccumulation processes.

The environmental impact of PFAS-related pollution is amplified by PFAS stability which leads to serious risks of bioaccumulation in animals belonging to the higher rankings in the trophic network, especially for aquatic organisms [11,12]. Recently, a European survey of persistent toxic substances in seafood has been reported evaluating potential threats to public health due to their assimilation [41]. For this reason, it is important to evaluate the bioaccumulative potential of such persistent pollutants as a function of the species, their diet, and living environment, considering both wild [12] and farmed species [46]. In this context, it is important to understand if PFAS contamination in a given species derives from a phenomenon of either direct (absorption or ingestion) or indirect (biomagnification) assimilation. It is, therefore, crucial to define the various indicators (and the differences among them) that allow for an overall assessment of the bioaccumulative potential. In fact, despite the unique definitions of such indicators by the European Chemical Agency [71] and other researchers in the field [72,73,74,75], the bioconcentration, bioaccumulation, and biomagnification factors are not always determined in the same research work, making it difficult to compare results between different studies. In this review, we are attempting to organize and discuss recent literature and compare results from different studies according to concepts illustrated in Figure 1 for bioconcentration (BCF), bioaccumulation (BAF), biota–sediment accumulation (BSAF), biomagnification (BMF), and trophic magnification (TMF) factors, and to the following definitions and equations.

Bioconcentration factor (BCF)

The BCF is intended to evaluate the uptake of a substance through chemical exposure, with the exclusion of dietary intake. The determination of BCF requires controlled conditions of exposure, is determinable in a laboratory, and takes into account only respiratory uptake which is governed by the kinetic constant k_Respiratory_. On the other hand, elimination processes (faecal egestion, metabolic biotransformation, gill elimination, and grow dilution) are taken into account by the cumulative elimination kinetic constant k_Elimination_. Thus, BCF is defined as the ratio between the kinetic constant of respiratory uptake and that of elimination (Equation (1)).

When the concentration of the chemical species in a specific tissue or specimen ([Substance]_Organism_) reaches a constant value (i.e., stationary state or plateau) after prolonged exposure to the chemical substance, due to the balance between the uptake and the elimination processes, BCF can be measured according to Equation (2) where: [Substance]_Organism_ is expressed as a “weight of substance”/“weight of wet sample” ratio using either mg/kg (ppm) [71] or g/kg units [72]; [Substance]_Water_ is the concentration of the chemical species freely dissolved in the water to which the organism is exposed (surrounding medium)—thus excluding precipitated or adsorbed chemicals in sediments—and expressed as a “weight of substance”/“volume of the medium” ratio using either mg/L (ppm) [71] or g/L units [72].

BCF (L/kg) = k_Respiratory_/k_Elimination_ (1)

BCF_SS_ (L/kg) = [Substance]_Organism_/[Substance]_Water_ (2)

Bioaccumulation factor (BAF)

Differently from BCF which requires controlled conditions and excludes the contribution of dietary intake, the BAF expresses the bioaccumulation of a substance in an organism through all possible routes of exposure, including diet, where the dietary uptake is governed by the kinetic constant k_Dietary_. Thus, BAF can be defined according to Equation (3) where [Substance]_Diet_, if known under controlled conditions, is the concentration of the investigated chemical in the diet, expressed as mg/kg. BAF can be measured, after a stationary state is reached, according to Equation (4). Under controlled laboratory conditions, the stationary state is reached when [Substance]_Organism_ reaches a plateau without further variations. For experiments in the field, the stationary state is assumed to be reached at the moment of monitoring. It is important to remark that, although measured through the same items (see Equations (2) and (4)) BAF and BCF cannot be determined in the same experiment since BCF excludes dietary intake of the investigated chemicals (i.e., animals are fed with uncontaminated food) while BAF considers dietary intake whether it is known under controlled conditions ([Substance]_Diet_) or unknown (in the field measurements). Differences between BCF and BAF are conceptually crucial since these two factors could be confused one for the other due to the similar way of determining them under stationary conditions [71,72,73,74,75].

BAF (L/kg) = (k_Respiratory_ + k_Dietary_ [Substance]_Diet_/[Substance]_Water_/k_Elimination_ (3)

BAF_SS_ (L/kg) = [Substance]_Organism_/[Substance]_Water_ (4)

Biota–sediment accumulation factor (BSAF)

Another, although less common, measurable potential of bioaccumulation in the field is the biota–sediment accumulation factor (BSAF). This descriptor expresses the relationship between the concentration of the test substance in the organism and the concentration of the test substance in sediments [72] and can be determined according to Equation (5)

BSAF = [Substance]_Organism_/[Substance]_Sediment_ (5)

Biomagnification factor (BMF)

Different from bioconcentration and bioaccumulation processes that involve a single type of tissue or organism, the biomagnification process refers to the increased concentration of a toxic chemical along the food chain, e.g., between prey and its predator. If the diet of the predator consists of only one type of prey, BMF can be determined, either in the field or under controlled conditions in the lab, according to Equation (6) as the ratio of the concentration of a substance in the predator organism [Substance]_Predator_ with that found in its prey [Substance]_Prey_ [73].

BMF = [Substance]_Predator_/[Substance]_Prey_ (6)

Additionally, by considering the definitions of BAF and BCF above, BMF could be evaluated also according to Equation (7) [73].

BMF = BAF_Predator_/BAF_Prey_ (7)

If the diet of the predator is variable, BMF can still be calculated under controlled conditions in the lab at the steady-state [71,72]. For instance, “dietary BMFs” may be determined in laboratory experiments according to Equation (8) by placing the monitored organism in uncontaminated water and exposing it to the investigated substance uniquely through the organism’s diet [75].

BMF = [Substance]_Organism_/[Substance]_Diet_ (8)

Conversely, under variable diet, the determination of BMF in the wild could be more difficult and, in these cases, the use of the trophic magnification factor (see below) would be more appropriate.

Trophic magnification factor (TMF)

While BMF considers biomagnification from one level of a trophic chain to the next higher level (i.e., contiguous species in the trophic network), TMF is used to identify the biomagnification process over an entire food chain or part of it [72]. In particular, empirical TMFs are deduced from field measurements, represent a weighted average of BMF over several trophic levels [75], and can be determined from the slope of log10[Substance]_Organism_ vs. the position (n) of the organism in the trophic chain or calculated according to Equation (9) [7,74].

Log_10_ TMF = (log_10_[Substance]_Organism n_ − log_10_[Substance]_Organism_ 1)/(n − 1) (9)

The determination of all of the above factors can be affected by choosing to determine concentrations using either wet or dried weights of the organism/tissue samples. Additionally, the tendency of organic chemicals to accumulate in lipid/proteic tissues should be taken into account by lipid/proteic normalization [75]. In the case of PFAS, which are considered proteinophilic substances, the proteic normalization can be performed by dividing the chemical concentration by the protein percentage of the animal or of the organ under examination [75,76].

If significant concentrations of a chemical substance are found in biota in remote areas, the above bioaccumulation descriptors can be used to assess the substance’s persistence, in particular for possible long-range transport.

In general, a substance fulfils the bioaccumulation criterion when the BCF or BAF in aquatic species is higher than 2000 (log_10_ BAF or log_10_ BCF > 3.3) and is considered as “very bioaccumulative” when the BCF or BAF exceeds 5000 (log_10_ BAF or log_10_ BCF > 3.7). On the other hand, with regard to BMF or TMF, values greater than 1 are considered significantly high [71].

### 3.1. Bioaccumulation Data

Many studies show that in strongly anthropized environments the above cited empirical descriptors meet the criteria to define PFCs as bioaccumulative substances [25,77] although, for specific substances such as PFOA, values of BCF and BAF are not always above the bioaccumulative threshold [71]. Since any of the bioaccumulative factors is determined based on a specific organism/tissue and can be evaluated in various geographical areas, it is important to analyse data from different studies to define the bioaccumulative potential of a specific polyfluorinated organic substance. For these reasons, in the following paragraphs, bioaccumulation studies are grouped based on the monitoring conditions as studies in the natural environment or under controlled experimental conditions.

#### 3.1.1. Bioaccumulation in the Wild

Since the bioaccumulative potential depends on the physicochemical properties, branched, linear, and differently functionalized PFCs show different affinities for tissues and organisms [33].

##### Bioaccumulation in Aquatic Flora

Despite the high bioaccumulation potential of PFCs and the intrinsic residential nature of the flora, there are only few PFCs bioaccumulation studies concerning either micro or macro aquatic flora. In one of these studies conducted in the Xiamen Sea area, the calculated BFC for kelp algae (*Thallus laminariae*) had a range of 2900–4600 L/kg for PFOS and 6700–14,300 L/kg for PFOA. For the latter, the calculated BCF was higher than that of the fauna investigated [78]. Conversely, lower BFC values (approximately 1000 L/kg) were found in the benthic algae of Michigan rivers (United States) [79]. Finally, high BAF values were calculated for floating plants in Lake Baiyangdian [80]. In particular, the log_10_ BAFs for PFOS, PFOA, PFNA and perfluorodecanoic acid (PFDA) were 3.0–4.1, 1.9–3.7, 2.9–4.4, and 3.2–4.2, respectively [80]. The values of the bioaccumulation factors of the above studies are similar to those calculated for aquatic fauna (see below) suggesting that PFCs may have similar (if not higher) bioaccumulation potentials for flora that should be further investigated.

##### Bioaccumulation in Aquatic Fauna

In general, long-chain PFCs have a higher bioaccumulative potential in biota than short-chain ones as observed in Asan Lake (South Korea) where PFDA and PFOS showed a log_10_ BAF value > 3.0 in fish species [81].

In the Xiamen Sea area, the BCFs calculated with the quantity of PFAAs in different trophic levels of aquatic animals ranged from 6400–9700 L/kg to 3300–8000 L/kg for PFOA and PFOS, respectively [78].

High BAF was determined also in wild crucian carp (*Carassius carassius*) collected from the Yubei River (China), with an average log_10_BAF values of 3.06 (in muscle) and 4.14 (in blood) for p-perfluorononenoxybenzenesulfonate (OBS) similar to the log_10_BAF values recorded for PFOS in the same species [82].

In a recent study considering 19 different PFAS, the analysis of seawater, sediment, and biota (*Ruditapes philippinarum*) allowed to determine both a log_10_BAF range of 2.53–4.32 and a log_10_BSAF range of 1.30–2.50 the Jiaozhou Bay coast area (China) [83]. Interestingly, these bioaccumulation factors correlated with the number of carbons in the PFAS chain with log_10_BAF increasing with the carbon chain length, whereas the log_10_BSAF values decreased with the carbon chain longer than C_8_ [83] since longer chained compounds would have a higher affinity for sediments.

A trophic transfer has also been detected in the Antarctic ecosystem. In particular, BMF values of perfluorobutyric acid (PFBA), perfluoroheptanoic acid (PFHpA), PFHxS, and PFOS between Archaeogastropoda and Neogastropoda ranged between 0.7 and 3.3 and indicated that short-chain PFAS may not be biomagnified along the food chain [84].

Biomagnification phenomena have been evaluated also in Gironde Estuary (France) based on PFCs concentrations found in mysids and copepods with BMF > 1 for PFOS, perfluorooctanesulfonamide (FOSA), and long-chain perfluorinated carboxylic acids (PFCAs) [85]. Trophic magnification was also proven in urban river environment (Orge, France) with values of TMFs > 1 for C_9_–C_14_ PFCA, C_7_–C_10_ PFSAs and several PFAAs such as sulphonated fluorotelomers 8:2 and 10:2) [86].

#### 3.1.2. PFAS Uptake under Controlled Experimental Conditions

Studies conducted under controlled conditions are important to understand which PFC is the most accumulated and to understand the differences in bioaccumulation between different species and tissues. In recent research work, some benthic fish (*Pseudogobius* sp.) were fed, for three weeks, with food contaminated with either PFOA, PFOS, or ammonium 2,3,3,3-tetrafluoro-2-(heptafluoropropoxy)propanoate (GenX). After a period of 42-day purification, the assimilation of PFOA was low (22%) corresponding to a low BMF of 0.021. Conversely, linear PFOS showed a much higher assimilation rate of 60% with BMF of 0.346, while GenX did not accumulate in the fish during exposure [65].

In zebrafish (*Danio rerio*) exposed to 10 μg/L of radiolabelled perfluorooctanoic acid (^14^C-PFOA), a BCF ranging between 20 and 30 was observed [87].

Zebrafish larvae exposed to three different concentrations of three PFAS, the determined BCFs were 113–193 for PFOS, 125–358 for F-53B, and 20–48 for OBS with different level of effects recorded on larvae’s development [88].

In amphibians (*Rana pipiens*, *Anaxyrus americanus*, and *Ambystoma tigrinum*) BCF value observed for PFOS (BCFs = 47–259) was greater than that recorded for PFOA (BCFs = 0.46–2.5) [89]. Similarly, in tadpoles of *R. pipiens* exposed for 40 days to three different concentrations of PFOS, PFHxS, PFOA, and 6:2 fluorotelomer sulfonate (6:2 FTS) the PFOS, showed higher accumulated levels with BCF ranging from 19.6 to 119.3 while other PFAS had BCF < 1.0 [66]. A similar trend was observed also for BAF and BSAF recently calculated on *R. pipiens* larvae where PFOS bioaccumulated at a higher rate than PFOA and where BSAF was up to two orders of magnitude lower than BAF [90].

Even higher bioaccumulation potential was observed in *Holothuria tubulosa* exposed to 6 PFAS at a concentration of 1 ppm where the log_10_ BAF ranged from 0.45 (for perfluorobutanoic acid (PFBuA) to 5.52 (for PFOS) in gonads and from 1.11 (for PFBuA) to 5.54 (for PFOS in the intestine). The log_10_ BSAF ranged from 1.1 (for PFOA) to 2.28 (for PFOS) in gonads and from 0.87 (for PFOS) to 3.56 (for PFOA) in the intestine [91].

As suggested by several protocols, generally the exposure times are about 28 days or until the steady-state is reached, this condition varies according to the elimination rate and half-life of the chemical in question.

Since the uptake of PFAS can be reached between 22 and 38 days, several organisms can accumulate these pollutants in the natural environments, considering a long time of exposure, thus creating potential problems of biomagnification and, in general, threatening the health of the ecosystems and organisms that are part of it.

As can be seen in the above studies, the values of bioaccumulation potentials calculated for field studies are generally higher than those obtained under controlled conditions. The reason for this difference may be that, unlike laboratory experiments, in nature lower concentration levels and longer exposure times favours the uptake of contaminants in organisms for which they have a higher affinity (compared to water). In this way, the organisms would be able to tolerate the increasing bioaccumulation of the pollutant, without reaching definitively the stationary state because of the open system of the environment in which they live.

These experiments, if integrated with molecular–biological approaches, could provide clear and exhaustive information about this emerging environmental threat. For example, recent studies of predictive toxicology based on innovative computational methods have evaluated the differences between some species in the binding affinity between per- and polyfluoroalkyl substances (PFAS) and liver fatty acid-binding protein (LFABP) finding similar PFAS bioaccumulation potentials for the investigated organisms. The evaluation showed that rainbow trout, humans, rats, and chickens have similar binding affinities for each PFAS, while medaka fish had a significantly weaker binding affinity for some PFAS [92]. It is therefore important to combine different types of experiments for the evaluation of bioaccumulation potentials and, in this context, molecular dynamics analysis opens the way of using computational tools to simulate or support studies where the sampling of rare or endangered species would be difficult.

## 4. Biomonitoring PFAS in Aquatic Biota

### 4.1. Biomonitoring PFAS in Aquatic Flora

Compared to the number of studies regarding the presence of the PFCs in the fauna (see below), biomonitoring data concerning aquatic flora are very few. One of these was conducted in Michigan rivers and showed that the benthic algae had concentrations of PFOS, FOSA, PFOA, and perfluorohexanesulfonic acid (PFHS) of: 2.6–3.1, <1, <0.2, and <2 ng/g, respectively [79]. Conversely, in the Xiamen Sea area, investigations on Kelp algae (*Thallus laminariae*) showed concentrations of 1.64–4.36 ng/g for PFOA and 1.42–3.58 for PFOS while the total PFAS concentration levels, [PFAS]_TOT_, ranged between 8.15 and 12.98 ng/g [78]. Finally, in the floating plants *Ceratophyllum demersum *L., *Hydrocharis dubia (Bl.) Backer*, and *Salvinia natans* collected in Lake Baiyangdian (China) high concentration levels of total PFCs (median value of 19.2 ng/g) were recorded with a prevalence of PFOA (max value of 10.4 ng/g) and PFNA (max value of 20.1 ng/g) followed by PFDA, PFOS, and perfluoropentanoic Acid (PFPeA), while PFHpA, perfluorobutanesulfonic acid (PFBS), PFHxS were not detected [80].

### 4.2. Biomonitoring PFAS in Aquatic Fauna

Aquatic organisms that live in contaminated environments (natural or artificial) tend to accumulate the pollutant more than water in which they live and in particular, PFAS in an aquatic organism can be transferred from the contaminated water, food or suspended sediment [81]. As already mentioned, although there may be a different biodistribution of the different PFAS in the different tissues and species, one of the common factors that play an important role in biota contamination lies in the geographical origin, of the analysed organisms. It is known that PFAS contamination levels of organisms caught in waters affected by anthropogenic pollution are generally higher than concentrations in organisms from open oceans [93,94,95]. This correlation is highlighted in an important mollusc aquaculture area Bohai Sea (China) where PFAS contamination was verified in different species of molluscs sampled in the various mussel farming with higher levels of contamination in samples taken near industrial areas [95].

For example, in the same sampling area located in the Cantabrian Sea (North Spain), near ports, sewage effluents, and wastewater, much lower total PFAS concentration levels [PFAS]_TOT_ were found in seawater (0.06 to 10.9 ng/L) compared to sediment (0.01–0.13 ng/g) and mussels (0.01–0.06 ng) [96]. Similarly, in the Orge river (France), the [PFAS]_TOT_ in seawater (73 ng/L) and in sediment (8.4 ng/g) were much lower than that found and in fish (*Leuciscus cephalus*) that ranged from 43.1 to 4997.2 ng/g; with a maximum log_10_ BAF values recorded for perfluorododecanoic acid (PFDoA) in the following order: plasma (6.7), liver (5.7), gills (5.7), gonads (5.5), and muscle (5) [97].

Another study near the wastewater area in Lake Tana (Ethiopia), showed that the averages of [PFAS]_TOT_ were 2.9 ng/L for surface water, 0.30 ng/g in surface sediment, and 1.2 ng/g in all fish species [98]. A similar trend was observed in Vietnam where [PFAS]_TOT_ in water (near discharge canal) was 107 ng/L. This represents the greatest recorded [PFAS]_TOT_ in these types of research, although remaining three orders of magnitude lower than [PFAS]_TOT_ in biota samples [99]. In the same way, in Jiaozhou bay coast (China) analyses of 35 PFAS carried out in seawater, sediment, and biological samples (*Ruditapes philippinarum*) showed, respectively, the following concentration range: 21.1–38.0 ng/L, 0.459 to 1.20 μg/kg, and 15.5–27.5 μg/kg [83].

In addition, significant differences in PFAS levels and composition profiles were found between the type of analysed samples. For example, in Lake Baiyangdian (China) the most abundant PFC in water was PFOA (1.70–73.5 ng/L), while in sediments and in aquatic animals the most abundant PFC was PFOS which was detected with a concentration range of 0.06–0.64 ng/g and 0.57–13.7 ng/g, respectively [80].

The levels of PFAS in wild sea bass *Dicentrarchus labrax* were higher (PFOS: 112–12,405 ng/kg; PFOA: 9–487 ng/kg) than those farmed. Interestingly, among farmed sea bass species, intensively farmed fishes showed lower PFAS values (PFOS: 11–105 ng/kg; PFOA: 9–51 ng/kg) compared to those extensively farmed [56].

This unexpected result was also supported by a study performed in 246 fishes and fishery products collected in various aquatic environments in the Netherlands where [PFAS]_TOT_ was higher in eels (43.6 ng/g) followed by shrimps (6.7 ng/g), marine fish (seabass) (4.5 ng/g), and farmed fish (e.g., trout, catfish, turbot, salmon, tilapia, pangasius) (0.06 ng/g) [100]. These studies agree with others where farmed fish showed lower PFAS contamination than freshwater or marine fish [101,102,103].

The reason for these results, which appear to contrast with the common expectation of a wild environment being less contaminated than a farmed habitat, may lie in the transport of pollutants through vectors by ocean currents or undetected sources of pollution. These transport phenomena can cause greater contamination in areas further away than those closest to industrial activities. For example, in the Antarctic ecosystem significant levels of PFAS have also been found in areas far from potential sources of contamination (e.g., [PFAS]_TOT_ = 4.97 ± 1.17 ng/g in Neogastropoda) [84].

Consistent with what was previously reported, for the complete assessment of the contamination status in an area, it is necessary to analyse the content of the pollutant in any the organisms or their environment, and to acquire other information about the ecosystem (biotic and abiotic components). However, since such monitoring could be difficult during the screening of wild environment, research should focus on one or more specific bioindicator species.

Ideally, the latter should respond to certain characteristics such as: being common and easy to sample, provide a high response in the presence of toxicants, show tolerance and resistance to environmental variability, and possess a reduced mobility—e.g., sessile species (for the evaluation of restricted spatial contamination), etc. [104,105]. In this way, although generally higher values are found in organisms living in the areas of the most contaminated areas, some species bioaccumulate more than others for example because the pollutant enters more easily into them for direct assimilation linked to their feeding behaviours (e.g., filter feeder) or for indirect assimilation (biomagnification) connected to their position (top) of the food chain (e.g., predators).

Indeed, PFAS bioindicator species already provide much information on the quality of an environment, and the concentrations found in their tissues reflect (albeit with much higher values) the levels of environmental contamination from PFAS [106].

Therefore, an overview of the [PFAS]_TOT_ values found in various aquatic organisms sampled in different countries is reported in Table 1.

The concentration levels of the PFAS correlate differently with the various tissues, based on the affinity of the individual PFAS for the different matrices, species due to the different intra- and interspecific physiological metabolic mechanisms of absorption and elimination of the pollutant.

On this basis, the values of bioaccumulation factors would provide a deeper insight of the affinity of a given PFC towards specific tissues. Not all the biomonitoring studies discussed above contain information about bioaccumulation factor and values reported in the literature are discussed below as a function of the type of tissues.

A value of BFC ranging between 8160–9680 L/kg for PFOA and 6430–7960 L/kg for PFOS was recorded in Grass carp muscle from the Xiamen freshwater area [78]. On the other hand, in saltwater, soft tissues of *Ocypode stimpsoni* showed a BCF of 6490–7440 L/kg for PFOA and 3270–4240 L/kg for PFOS, while in soft tissues of *Ostrea gigas* BCF ranged between 6410–9680 (PFOA) and 4180–6430 L/kg (PFOS) [78]. In Jiaozhou Bay (China) soft tissues of saltwater clam *(Ruditapes philippinarum*) showed log_10_BAF between 2.53–4.32 for all 19 types of PFAS detected with values positively correlated with carbon chain length (C_8_–C_13_) [83]. Conversely, high values of log_10_BAF were calculated for small crustacean (mysids and copepods) in France estuarine area. In particular, in the whole body, the highest value was detected for L-FOSA (4.1 for copepods and 4.9 for mysids), confirming the great threat of PFCs due to their bioaccumulation (and biomagnification) potential also in small organisms [85]. Conversely, a biomonitoring screening conducted on different aquatic organisms and tissues in Vietnam, reported that BCF values in seven fish species were always higher in the liver (max BCF value found in tilapia for heneicosafluoroundecanoic acid (PFUnDA) of 142,764 L/kg followed by PFDA of 22,953 L/kg, PFDoA of 7729 L/kg, and PFOS of 3551 L/kg) than in the muscles (max BCF value found in tilapia for PFUnDA of 9169 L/kg) and the whole body (max BCF value found in dusky sleeper for PFNA of 1627 L/kg) [99]. On the other hand, in the same study, BCF values were higher in bivalves’ soft tissues (max BCF value found in golden freshwater clam for PFHxS of 2781 L/kg) than in crustaceans (max BCF value found in paddle crab for PFHpA of 1523 L/kg) and gastropods (max BCF value found in golden applesnail for PFHxS of 1606 L/kg) [99]. These reported bioaccumulative potential data are coherent with the evidence emerged in the recent review of Burkhard [25] where differences in BFC values between different PFCs, aquatic organisms and tissues are reported.

## 5. Biodistribution and Contamination Profile

The distribution and contamination profile of PFAS in the aquatic organism is consistent with the results of previous studies, which showed that the distribution can be affected by different variables such as environmental contamination specific metabolism and diet [98,115,116]. Therefore, besides differences due to geographical location, it is important to remark that biodistribution studies must compare species as close as possible to each other, since the diet, and physiological mechanisms are intrinsically different between different species.

### 5.1. Contamination Profile

A study comparing fishes with different nutritional behaviour showed that herbivorous and omnivorous fishes, such as *Labeobarbus intermedius*, *Oreochromis niloticus*, and *Clarias gariepinus*, contained a higher proportion of short-chain PFAS with respect to piscivorous fishes *Labeobarbus megastoma* and *Labeobarbus gorguari*. The latter, however, contained an overall higher total PFAS concentrations and a higher proportion of long-chain PFAS with respect to non-piscivorous species [98]. Conversely, a higher percentage of long-chain PFAS has been recorded for the PFCs contamination profile in the plasma of the threatened herbivore manatee of the West Indies (*Trichechus manatus*) [117]. The contamination profile could change also depending on the sampling site, even when belonging to the same area. For instance, a study on molluscs from the semi-closed basin of the Bohai (China) and investigating the presence of 23 PFCs, showed differences between aquaculture sites and more restricted areas with PFOA being the more abundant component (87% of the total PFAS) showing a very high frequency of detection (i.e., percentage of samples where PFOA has been detected) of 98%, followed by PFNA, perfluorodecane sulfonic acid (PFDS) and PFOS [95]. Indeed, the most frequently detected PFCs in various tissues of aquatic organisms are long-chain PFAS such as PFOS [56,58,115,117,118,119] and PFOA [78,83,95]. This frequency is particularly important when referred to organisms that are directly consumed by humans or by endangered species. A worryingly study focusing on the analysis of 11 PFAS in different edible fish species sampled in South Carolina (mullet, croaker, spot, red drum, seatrout, and flounder), showed that the concentrations of PFOS were between 25.5–69.6% of the total detected PFAS, with values exceeding the threshold limits allowed for food consumption [110].

The concentration levels of a specific PFC in the different tissues of the same species (e.g., PFOA in the liver of species A vs. PFOA in plasma of species A) are usually correlated. However, this correlation may lack for the same PFC analysed in the same organs of different species (e.g., PFOA in the liver of species A vs. PFOA in the liver of species B). This may be partially due to the differences in species and tissue-specific proteins, circulation mechanisms, depuration pathways, and feeding behaviour among the species [115]. For example, PFOS accounted for 87.6% of the total PFCs in minnow eggs, whereas it only accounted for 42.6% of the total fluorinated compounds in white shrimp eggs [115]. The same also applies to phylogenetically close species, and for [PFAS]_TOT_, in a recent work, for example, considering the same marine area but different species (of mollusc) analysed, [PFAS]_TOT_ showed the following trend: clams > mussels > scallops > peels > oysters [95].

In general, individuals of the same species from different sampling sites should accumulate PFAS in the same proportion thus reflecting PFAS contamination of their local habitat. For example, in crab *Ocytopode stimsoni* the ratio between PFOA and PFOS found in tissues was the same as that found in water and sediment from the two different sampling areas in Xiamen [78].

However, this correlation between PFCs contamination in the organism and its habitat does not always occur since a given organism could be selective in the type of uptaken PFAS thus presenting a different contamination profile compared to the collection site. For example, despite the predominance of PFOS compared to PFOA in water and sediments of two sampling areas of Xiamen, the oyster *Cassostrea gigas* shows higher PFOA concentration levels than PFOS) [78]. Similarly, in biomonitoring research conducted in Vietnam, the concentrations of PFOA and PFOS in water were dominant compared to the other 11 PFAS; however, in the biological samples of various organisms analysed, PFUnDA showed to be more present, in particular in the liver of the tilapia fish [99]. Additionally, in the west coast of Korea, biomonitoring screening on fish showed higher PFAS levels than crabs, gastropods, and bivalves; however, the different affinity of PFHpA and PFOS for the organism did not allow to reproduce in the organism the same contamination profile of the environment due to different bioaccumulative factors of the investigated PFCs [120]. For this reason, it is very important to validate any biomonitoring study by determining the significance of the correlation between the level of contamination (e.g., total PFAS) of the organism as a signal for environmental contamination and the profile of contamination (e.g., the contribution of each PFC to the contamination). While a contaminated organism is generally a reliable indicator of a contaminated site, the profile of contamination of the organism would represent that of the environment only in the case of similar bioaccumulative factors of the different contaminants.

### 5.2. Tissues Biodistribution

**Figure 2:**
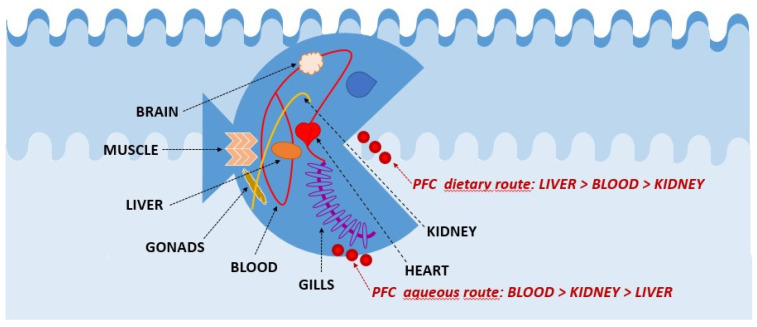
Scheme of preferred tissues for the biodistribution of PFCs in fish.

Besides bioaccumulating differently between species, PFCs are also variably distributed among the different tissues of the same organism generally based on the type of PFC intake. It is noteworthy that in exposure experiments through contaminated food, the highest concentration levels were recorded in the liver followed by blood and kidneys; while in aqueous exposure studies, higher concentrations were found in the blood followed by the kidneys and liver [65] (Figure 2).

In a study on sharks, PFASs were found at different concentration levels in the following order: gonads > heart > liver > gills > muscle [42,99]. Likewise, in European chub (*Leuciscus cephalus*) tissues, PFCs accumulation level followed the order: plasma > liver > gills > gonads > muscle [97]. In Danjiangkou reservoir and Hanjiang river (China), different fish species and tissues were analysed showing a higher concentration of PFAS in the liver and egg than in the muscles of the same individuals [111]. Additionally, in Vietnam, two species of fishes showed greater contamination of PFCs in the liver rather than in the muscles [99]. Similarly, PFAS contamination in various tissues of fish caught from lakes in the Alpine area was observed to be at a higher concentration in the blood and liver with respect to than found in the muscles [23]. In Taihu Lake (China) an investigation of different tissues of various aquatic organisms showed the highest concentration of PFASs in the liver and eggs while the lowest was in muscle [115].

In an experiment on zebrafish exposed to ^14^C-PFOA, autoradiograms confirmed the highest labelling of PFOA in bile, intestine (an enterohepatic circulation of PFOA), and in the maturation of vitellogenic oocytes [87].

Within the same organism, the pollutant is distributed differently in the different tissues, influencing their physiological conditions and often leading to biomolecular effects that negatively affect the individual. Therefore, biodistribution studies represent a major concern for the health of exposed animals since most of the organs or tissues having the major affinity for PFCs accumulation are involved in vital functions.

## 6. Effects of PFCs Exposure

The effects of perfluoroalkylic emerging pollutants are worrying since they involve the functionality of several organs and are generally more severe with increasing time of exposure and concentration of the contaminant [8,26,38,121]. Compared to the effects of PFCs studied in humans, the biomolecular effects shown in aquatic organisms are more subject to controlled laboratory conditions, derive from a variety of experiments, and thus represent a wider panorama of possible consequences to PFCs exposure [16,17,87,122,123,124,125,126,127,128,129,130,131,132]. Furthermore, although the human or mammalian model in general [18] may be far from the other animal models considered for these experiments, it is also known that aquatic organisms have long provided valuable information for the study of basic biological processes [133] showing also biomolecular responses similar to humans [134].

In addition, research on aquatic organisms is useful for safeguarding the balance of the ecosystems and for evaluating adverse and potential effects (e.g., biomolecular, physiological, toxic: cytotoxic, genotoxic, embryotoxic, etc.) on all species that are part of the studied trophic network.

In this context, it is important to analyse the data concerning the toxicity descriptors, EC_50_, LC_50_, and IC_50_, defined below.

An EC_50_ (effective concentration) an estimate of the concentration of a toxic substance that is required to produce an observed effect (endpoint) in 50% of the group of organisms exposed to the substance. An LC_50_ (lethal concentration) is a special case of the EC_50_ in which the recorded effect or endpoint is the death of the organism [135,136].

Finally, the IC_50_ (inhibitory concentration) is the concentration of the chemical compound required for the inhibition of the process or biological component of 50% of the group of organisms exposed to the substance [135].

On these bases, all the effects reported below have been organized and presented as a function of the studied aquatic organisms.

### 6.1. Algae

The primary producers in the trophic network are usually plants and algae that constitute the first level of the food chain. Therefore, it is important to verify the effects of perfluorinated compounds in plant or algal organisms. In fact, besides consequences on the same plant organisms, bioaccumulation of PFCs in plants and algae can have an impact on the individuals who feed on them or onto any organism linked with their system of ecological relationship. Parameters to be considered when assessing ecotoxicity in macrophytes can include root elongation rates, growth inhibition, and photosynthesis.

For instance, the aquatic toxicity of seven PFAS (PFBA, 2,2,3,3,4,4,5,5-Octafluoro-1-pentanol (5H 4:1 FTOH), PFOA, PFNA, PFDA, perfluoroundecanoic acid (PFUnA) and PFDoA) was observed to determine the effect on the elongation of the roots of the seeds of *Lactuca sativa* (EC_50_ range: 0.14–4.19 mM) and on the photosynthesis of *Pseudokirchneriella subcapitata* (EC_50_ range: 0.39–4.85 mM). Results showed that toxic effects are more severe with the length of the fluorinated carbon chain except for PFBA in *P. subcapitata* [137].

A similar correlation emerged in a toxicity test on species representative of the algal flora of the Baltic Sea (*Chlorella vulgaris*, *Skeletonema marinoi*, and *Geitlerinema amphibium*). In particular, the growth inhibition effect of PFCs (perfluorohexanoic acid (PFHxA), PFHpA, PFOA, and PFNA) at 72 h expressed as EC_50_ values ranged from 0.28 mM to 12.84 mM [14].

Toxicity test of PFOS on green algae *Selenastrum capricornutum* and *Chlorella vulgaris*, the floating macrophyte *Lemna gibba*, was performed to observe autotroph inhibition of growth. The NOEC (No Observed Effect Concentration) values were 5.3, 8.2, and 6.6 mg/L for *S. capricornutum*, *C. vulgaris*, and *L. gibba*, respectively. The most sensitive organism based on IC_50_ was *L. gibba*, with IC_50_ value of 31 mg/L [138]. Similarly, in microalga *Isochrysis galbana* acute EC_50_/72 h value for PFOS was 37.5 mg/L instead for PFOA was 163.6 mg/L [16].

The observed toxicological threshold is higher than concentrations of PFCs recorded in the aquatic media which usually range from few units of ng/L in less contaminated site to hundreds of ng/L in contaminated sites [6,139]. However, this finding should not promote a careless dispersion of PFCs pollutants in the environment, since PFCs have shown a very high bioaccumulative potential which increases the actual concentration in the organism by several order of magnitude compared to the environmental concentration.

### 6.2. Invertebrates

In recent decades, aquatic invertebrates have received increasing attention for their use as research models to study the effects of various types of toxic substances. In addition, their use constitutes a valid alternative that minimizes ethical concerns and offers the opportunity to observe behaviours, anatomy, physiological principles, pathologies, results of genetic manipulation, and mechanisms of pharmacological actions [140].

Genotoxic effects of PFOS, PFOA, PFNA, and PFDA were investigated on marine mussels (*Perna viridis*) showing that exposure could damage the organism’s genetic material to varying extents, including DNA strand breaks, fragmentation, and apoptosis. In particular, PFOS exhibited higher genotoxicity than the other tested compounds. Although primary DNA damage was shown to be recoverable after exposure ceased, permanent genetic damage caused by elevated PFCs concentrations was not restored [124].

In sea urchins *Glyptocidaris crenularis*, exposure to PFOS (followed by a period of purification) resulted in decreased motor skills, nutrition, and dropped spines. Furthermore, it also modified the activity of superoxide dismutase in the coelomic fluid and the activity of methylation and demethylation catalases in the gonad [126].

Toxicological descriptors EC_50_ and LC_50_ in embryos of *Paracentrotus lividus*, showed high embryotoxicity of increasing concentrations of PFOS and perfluorooctanesulfonyl fluoride (POSF). Indeed, low concentrations of these PFCs caused malformations in the skeletal system and high concentrations inhibited the growth of embryos in the early life stages. As a result, POSF (EC_50_/72 h was 1.074 mg/L) was more toxic than PFOS (EC_50_/72 h: 1.795 mg/L) [17]. In another study on *P. lividus* embryos, and with different biological effects taken into consideration (retarded plutei, skeletal malformations; blocked gastrula or blastula [17] vs. growth inhibition [16]), and EC_50_/96 h for PFOS (20 mg/L) and PFOA (110 mg/L) was determined [16], while EC/LC_50_/96 h levels for PFOS and PFOA were 1.7 mg/L and 19 mg/L, respectively, in sea urchin embryos of *Strongylocentrotus purpuratus* [141]. Similarly in crustacean *Siriella armata* Acute EC_50_/96 h values for PFOS was 6.9 mg/L and for PFOA was 15.5 mg/L [16], while in another mysid species *Americamysis bahia* the EC/LC_50_/96 h levels for PFOS and PFOA were 5 and 24 mg/L, respectively [141].

Regarding the development of *Mitilus galloprovincialis* embryos, the EC_50_/96 h was 1.1 mg/L for PFOS and 12 mg/L for PFOA; while regarding survival, the LC_50_/48 h values were 1.07 for PFOS and 9.98 for PFOA [141].

Furthermore, genotoxic effects in adults of *Mytilus edulis* exposed to PFOA were recorded by the significant alteration of the activity of antioxidant enzymes and numerous processes including those related to lipid metabolism, amino acids, and carbohydrates [142].

Toxic effects of PFOA and PFOS have also been observed in freshwater species such as *Dugesia japonica*, *Physa acuta*, *Daphnia magna*, and *Neocaridina denticulate*. In the latter study, PFOS was more toxic than PFOA, with an LC_50_/96 h ranging from 23 to 178 mg/L while PFOA had LC_50_/96 h ranging from 337 to 672 mg/L. The most sensitive freshwater species to PFOS was *N. denticulate* (LC_50_/96 h of 10 mg/L) while *P. acuta* showed the greatest resistance to exposure [123].

Small invertebrates (such as the aforementioned *Daphnia magna*) form the basis of the food chain and can contaminate the entire trophic network through the phenomenon of biomagnification. In addition, the experiments conducted on these organisms are important to evaluate survival rate under stress conditions and to analyse the biological effects of PFCs in easy-to-manage model systems. For example, in midge larvae (*Chironomus riparius*) exposed to sediments containing various PFAS bioaccumulation has been observed mainly during the fourth instar larvae exponential growth phase [143]. Concerning the effects deriving from exposure to PFOS and PFBS, *C. riparius* has shown that direct mutagenicity or induced stress conditions can be the basis for an increase in the mutation rate with negative evolutionary consequences [125].

In rotifers, reproductive bioassays indicated that exposure to PFOS and PFOA inhibited the growth of organisms, their production and hatching of eggs [144].

In *D. magna*, the aquatic toxicity of a fluoroalkylated polymer and perfluoroalkyl carboxylic acids (PFBA, PFHxA, PFOA) showed that the acute toxicity decreased with decreasing carbon chain length, although the polymer did not show a dose-related effect [128]. Similarly, in *Daphnia carinata*, exposed to various concentrations of PFOA and PFOS, acute (48 h) and chronic (21 days) toxicity tests showed that PFOS expresses greater toxicity causing, among others, effects of inducing gene aberrations. In particular, the LC_50_/48 h values for PFOA and PFOS were 78.2 mg/L and 8.8 mg/L, respectively. Additionally, chronic exposure to PFOS at a concentration of 0.001 mg/L showed effects of mortality and reproductive defects [4]. As seen in algae for growth reduction (IC_50_), the endpoints of a toxicity experiment can be of different types. Therefore, also in other organisms, studies must be based on the most representative response arising from the biological process more sensitive to PFC-related stress. For example, in the bioluminescent dinoflagellates *Pyrocystis lunula* treated with PFOA (EC_50_/24 h: 18 mg/L) and PFOS (EC_50_/24 h: 4.9 mg/L), the chosen endpoint was a lower emission of light (bioluminescence output) compared to a control corresponding to an adverse response of the organism to chemicals [141].

### 6.3. Fishes

Laboratory studies on the toxic effect of PFAS related to their bioconcentration and specific tissue distribution in fish, may be useful for predicting the persistence and environmental effects of chemicals. Moreover, these studies allow the creation physiologically based pharmacokinetic (PBPK) model that incorporates the biotransformation of the PFCs precursors and simultaneously predicts the distribution of the precursor and its metabolites in the different tissues.

An example of PBPK for PFCs in rainbow trout (*Oncorhynchus mykiss*) was the formation of PFOA from the biotransformation of 8:2 fluorotelomer carboxylic acid (8:2 FTCA) [131]. Regarding the negative effect of PFCs in fish, exposure of Chinook salmon (*Oncorhynchus tshawytscha*) to a mixture of 16 substances, including 3 perfluorinated compounds (PFDA, PFOS, FOSA), resulted in disorders on the mitochondrial function of the liver and its physiological processes [130].

Similarly, another study on the evaluation of the cytotoxic and genotoxic effects of PFDoA, on the Japanese medaka fish, *Oryzias latipes*, showed that subchronic exposure to such compound caused DNA damage with simultaneous induction of different erythrocyte abnormalities [132].

Similarly to invertebrate studies, in *Psetta maxima*, PFOS acute EC_50_ values (0.11 mg/L) were higher than PFOA toxicity with EC_50_ = 11.9 mg/L [16].

In zebrafish embryos, sublethal exposure to PFOS, PFNA, and PFOA caused a decrease in the total body length, increased the expression of a muscle development regulating gene (*tfc3a*) while decreasing the expression of genes involved in protein transport (*ap1s*), hyperactive locomotor activity and behavioural changes that remained permanent in adults [129].

These results are reasonably connected to the chemical accumulation through yolk proteins in oocytes [87], with consequent plausible risks of adverse effects on early embryonic development and offspring health. PFOS and PFOA inhibited embryo development and caused embryo abnormality and death [122]. Moreover, PFOA and PFOS inhibited the growth of zebrafish liver cells line and increased the percentage of cell apoptosis significantly with the treatment [127].

Conversely, zebrafish larvae exposed to F-53B, PFOS, and OBS, increased energy expenditure, reduced feed intake (as a function of concentration), and, except for OBS, the expression of genes involved in metabolic pathways at the transcriptional and translational level [88]. Interestingly, molecular docking computational studies revealed that the binding affinities of PFAS to glucokinase decreases following the order F-53B > PFOS > OBS. Experimental findings confirmed that F-53B has the highest potential for bioconcentration and the strongest metabolic disturbing effects [88].

Additionally, exposure to perfluoropolyethers (PFPeS), PFHxS, perfluoroheptanesulfonic acid (PFHpS), or PFOS resulted in a shared toxicity phenotype characterized by body axis and swim bladder defects, as well as hyperactivity [145]. Interestingly, the observed neurotoxicity of the development is directly proportional to the increase in the length of the carbon chain [145]. For instance, in another PFAA-exposure study in zebrafish embryos/embryonic cells, sulfonic acid toxicity was greater than carboxylic acid one, with a mortality rate that increased with carbon chain length (PFOA > PFHxA ≫ PFBA) [146]. This finding is in agreement with previous studies showing an LC_50_ (48 h exposure) of 1005 mg/L for PFOA and of 107 mg/L for PFOS [122].

Interestingly, while exposure of zebrafish embryos to either PFOS or PFOA showed higher toxicity of PFOS, the exposure to a mixture of the two PFCs showed complex interactive effects that varied from additive to synergistic, antagonistic, and, lastly, again to synergic effect, by increasing the molar ratios of PFOS [147].

### 6.4. Amphibians

Another class of organisms widely used in laboratory tests is represented by amphibians. These organisms are excellent models for the study of diseases due to their easy management, breeding, high resistance, and *human-like* genetic context. Moreover, these models are particularly useful when the impact of high doses of environmentally relevant PFCs needs to be investigated.

For example, in a study on the PFCs influence on the nervous system of the Northern leopard frogs (*Lithobates pipiens*) it was observed that PFCs should be evaluated for a potential role in diseases that target dopamine production. In detail, dopamine levels decreased significantly in the brains of frogs treated with PFOA (1000 ppb) and PFOS (100 and 1000 ppb) [148].

A recent study has been conducted also for skin exposure to PFCs, occurring through contact with dry moss containing PFAS at 0, 80, 800, and 8000 ppb. The survival and growth of toads (*Anaxyrus americanus*), salamanders (*Ambystoma tigrinum*), and frogs (*Rana pipiens*), and effects on the final length of the muzzle (SVL), on the mass index of the scale (SMI), and the measurements of the relative body conditions, have been demonstrated and varied by species and compound [149].

Serious disorders in the liver and heart in *Xenopus* embryogenesis were observed during the evaluation of developmental toxicity and teratogenicity of PFHxA and PFHpA. PFHpA, which has one additional carbon atom with respect to PFHxA, produced more serious effects by increasing the phosphorylation of the signal-regulated extracellular kinase (ERK) and the N-terminal c-Jun (JNK) [150]. Conversely, tadpoles of *Xenopus laevis* exposed to increasing concentrations of PFOS and PFBS showed hepatohistological effects at concentrations of 100 and 1000 μg/L. Despite the absence of significant effects on survival and growth, PFOS caused spermatogonial degeneration [151], and administering PFOS, PFHxS, PFOA, 6:2 FTS at different concentrations in tadpoles of *Rana pipiens* resulted in developmental delays [66].

## 7. Critical Aspects and Future Environmental Implications

The negative impact of PFCs in the aquatic environment is a major concern for the entire ecosystem that relies on the delicate equilibria between water, bed, shoreline, and the species living in them. The effect of PFCs contamination depends on the exposure conditions (currents, temperature, exposure time, concentration, etc.) and on the physiological mechanism of the affected organism. By comparing taxonomically different aquatic organisms, it is remarkable that micro-and macrophytes showed the highest resistance to effects caused by PFCs exposure. Algae are more prone to survive bioaccumulative processes and are therefore the most suitable resident organisms for biomonitoring studies. Nevertheless, their survival rate does not exempt them from suffering negative effects of PFCs exposure, such as growth inhibition or development anomalies. Moreover, once contaminated, the restoration of a pristine state depends on the elimination time which, for instance, is generally longer for long-chained PFAS.

Conversely, in the animal kingdom, the effect of PFCs exposure includes, among others, DNA damage, aberrations in gene expression, endocrine system disruption, morphological and functional anomalies. When exposure involves fish or echinoderms embryos, such effects are worsened and lead to growth inhibition, developmental anomalies, and death. Tolerance toward PFCs exposure increases with the size of the exposed organisms, either among different species or within individuals of the same species. This is particularly true when individuals of different sizes are exposed to the same concentration of the contaminant, due to either size (or growth) dilution phenomena or to changes in toxicokinetics related to ontogenesis.

In this context, the high bioaccumulation factors and the negative effects recorded after prolonged exposure to low concentrations of PFCs pose serious questions about the assessment of the limit of tolerable PFCs contaminations indicated in environmental regulations. Additionally, some of the effects could be underestimated, especially in the case of emerging pollutants, if ecotoxicological studies are limited to a few conditions or species. A high variability of bioaccumulation and toxic effect has been observed among individuals of different species and upon variation of exposure conditions (e.g., contaminated water, contaminated food, or contaminated sediments).

The results of bioaccumulation studies along species belonging to the same food chain are useful to highlight those species that are less prone to bioconcentrate the monitored substance, thus suggesting strategies for preferential consumption. However, due to the variability of environmental conditions and type of exposure (acute, chronic, diffuse, localized, etc.), the comparison of results arising from different studies requires careful data treatment or normalization.

Despite the negative impact of PFCs has prompted regulatory limitations to their use and production, the development of new alternative PFCs poses new potential environmental threats. In these cases, ecotoxicity studies performed under controlled conditions on animal models are crucial to evaluate all the risks of the use of alternative PFCs.

In this frame, contrast measures to PFCs related pollution are emerging among biodegradation studies taking into consideration PFAS degrading microorganisms. Additionally, alternative policies to production limitations are those involving waste management both at the industrial and at the consumer level, since the best pollution prevention is to avoid dispersion of PFCs containing materials in the environment.

## Figures and Tables

**Table 1:** Number of monitored PFCs (PFAS) and their total concentration as a function of investigated species in different locations in wild environment.

Fish	Location in Wild	Samples Analysed	^1^ [PFAS]_TOT_ (ng/g)	Ref.
Red seabream (*Pagrus major*)	China Seawater	Muscle	Σ9 PFAS: 0.04–2.14	[107]
Chameleon goby (*Tridentiger trigonocephalus*)	China Seawater	Muscle	Σ6 PFAS: 10.97–12.93	[78]
Baltic cod (*Gadus morhua*)	Baltic Sea Seawater	Liver	Σ28 PFAS: 6.03–23.9	[108]
Shortfin mako shark (*Isurus oxyrinchus*)	Greece Seawater	Muscle, Gills, Heart	Σ15 PFAS: 3.2–10.3	[109]
Angular roughshark (*Oxynotus centrina*)	Greece Seawater	Muscle, Liver	Σ15 PFAS: 17.9–85.1	[109]
Giant devil ray (*Mobula mobular*)	Greece Seawater	Muscle, Gills	Σ15 PFAS: 1.5–4.4	[109]
Smalltooth sand tiger (*Odontaspisferox*)	Greece Seawater	Gills, Liver	Σ15 PFAS: 62.2–65.4	[109]
Bigeye thresher (*Alopias superciliosus*)	Greece Seawater	Muscle, Gills, Liver, Heart	Σ15 PFAS: 3.1–48.1	[109]
Sharpnose sevengills shark (*Heptranchias perlo*)	Greece Seawater	Muscle, Gills, Liver, Gonad, Heart	Σ15 PFAS: <LOQ-35	[109]
Bluntnose sixgills shark (*Hexanchus griseus*)	Greece Seawater	Muscle, Gills, Liver, Gonad, Heart	Σ15 PFAS: 1.1–66.3	[109]
Blue shark (*Prionace glauca*)	Greece Seawater	Muscle, Gills, Liver, Heart	Σ15 PFAS: 0.3–15.5	[109]
Atlantic croaker (*Micropogonias undulatus*)	South Carolina Seawater	Whole fish	Σ11 PFAS: 15.2–21.3	[110]
Red drum (*Sciaenops ocellatus*)	South Carolina Seawater	Whole fish	Σ11 PFAS: 11.3–66.1	[110]
Spot (*Leiostomus xanthurus*)	South Carolina Seawater	Whole fish	Σ11 PFAS: 14.7–67.8	[110]
Spotted seatrout (*Cynoscion nebulosus*)	South Carolina Seawater	Whole fish	Σ11 PFAS: 17.3–85.4	[110]
Striped mullet (*Mugil cephalus*)	South Carolina Seawater	Whole fish	Σ11 PFAS: 6.2–20.7	[110]
Yellow croaker (*Larimichthys polyactis*)	China Freshwater	Liver, Muscle	Σ8 PFAS: 8.99–87.9	[111]
Mandarin fish (*Siniperca chuatsi*)	China Freshwater	Liver, Muscle, Eggs	Σ8 PFAS: 3.02–51.2	[111]
Crucian Carp (*Carassius carassius*)	China Freshwater	Muscle	Σ8 PFAS: 3.15–4.09	[111]
Crucian Carp (*Carassius carassius*)	South Korea Freshwater	Muscle	Σ19 PFAS: 17.6 ± 10.0	[81]
Common Carp (*Cyprinus carpio*)	South Korea Freshwater	Muscle	Σ19 PFAS: 50.6 ± 71.6	[81]
Grass carp (*Ctenopharyngodon idellus*)	China Freshwater	Muscle	Σ6 PFAS: 8.87–10.66	[78]
Barbel steed (*Hemibarbus labeo*)	South Korea Freshwater	Muscle	Σ19 PFAS 16.7 ± 2.7	[81]
Bass (*Micropterus salmoides*)	South Korea Freshwater	Muscle	Σ19 PFAS: 40.3 ± 13.7	[81]
Bass (*Micropterus salmoides*)	China Freshwater	Muscle	Σ8 PFAS: 3.02	[111]
Bluegill (*Lepomis macrochirus*)	South Korea Freshwater	Muscle	Σ19 PFAS: 32.4 ± 11.0	[81]
Skygager (*Chanodichthys dabryi*)	South Korea Freshwater	Muscle	Σ19 PFAS: 30.5 ± 25.3	[81]
Tilapia (*Oreochrommic niloticus*)	Vietnam Freshwater	Liver, Muscle	Σ13 PFAS: 0.5–10.6	[99]
Stripped snakehead (*Chana striata*)	Vietnam Freshwater	Liver, Muscle	Σ13 PFAS: 0.18–1.01	[99]
Dusky sleeper (*Eleotris fusca*)	Vietnam Freshwater	Whole body	Σ13 PFAS: 0.92	[99]
Shark catfish (*Pangasius elongatus*)	Vietnam Freshwater	Whole body	Σ13 PFAS: 0.3	[99]
Flying barb (*Esomus danricus*)	Vietnam Freshwater	Whole body	Σ13 PFAS: 0.91	[99]
Ninespine stickleback (*Pungitius pungitius*)	Alaska Freshwater	Whole body	Σ31 PFAS: 3.66–15.6	[112]
European eel (*Anguilla anguilla*)	Netherlands Freshwater	Muscle	Σ16 PFAS: 4.7–172	[100]
**Crustacea**				
Ghost crab (*Ocytopode stimpsoni*)	China Seawater	Soft tissues	Σ6 PFAS: 7.8–10.47	[78]
Hermit crab (*Clibanarius infraspinatus*)	China Seawater	Soft tissues	Σ6 PFAS: 7.73–8.06	[78]
Asian paddle crab (*Charybdis japonica*)	Vietnam Freshwater	Soft tissues	Σ13 PFAS: 0.61	[99]
Giant prawn (*Macrobrachium rosenbergii*)	Vietnam Freshwater	Soft tissues	Σ13 PFAS: 0.24–0.58	[99]
Shrimp (*Palaemon longirostris*)	France Estuarine areas	Whole body	Σ22 PFAS: 4.5 ± 1.2	[85]
Brown shrimp (*crangon crangon*)	France Estuarine areas	Whole body	Σ22 PFAS: 11 ± 2	[85]
Mysid shrimps (*Mysidacea, ind.*)	France Estuarine areas	Whole body	Σ22 PFAS: 7.2 ± 2.0	[85]
Copepods (*Copepoda, ind*.)	France Estuarine areas	Whole body	Σ22 PFAS: 2.9 ± 0.8	[85]
Zooplankton (*Copepoda, Cladocera*)	Italy Freshwater	Whole body	Σ12 PFAS: 7.6	[113]
**Mollusca**				
Shell fish (*Ruditapes philippinarum*)	China Seawater	Soft tissues	Σ19 PFAS: 15.5–27.5	[83]
Oyster (*Cassostrea gigas*)	China Seawater	Soft tissues	Σ6 PFAS: 12.45–12.76	[78]
Quagga mussels (*Dreissena bugensis*)	Belgium Freshwater	Soft tissues	Σ15 PFAS: 21.88	[114]
Asian clam (*Corbicula fuminea*)	Belgium Freshwater	Soft tissues	Σ15 PFAS: 20.79	[114]
Golden clam (*Corbicula fluminea*)	Vietnam Freshwater	Soft tissues	Σ13 PFAS: 0.73	[99]
Golden apple snail (*Pomacea canaliculata*)	Vietnam Freshwater	Soft tissues	Σ13 PFAS: 0.22–0.6	[99]
**Mammalian**				
Killer whales (*Orcinus orca*)	Greenland Seawater	liver	Σ36 PFAS: 614 ± 49	[58]
Harbor seals (*Phoca vitulina*)	Sweden Seawater	liver	Σ36 PFAS: 640 ± 51	[58]
Ringed seals (*Phoca hispida*)	Sweden Seawater	liver	Σ36 PFAS: 536 ± 43	[58]

^1^ Values (ng/g) reported the total PFAS concentration indicated as average, average ± standard deviation, or min–max concentration ranges. The symbol Σ precedes the number of PFAS contributing to the total concentration.

## Abbreviations

Perfluorohexanesulfonic acid (PFHS); 2,2,3,3,4,4,5,5-Octafluoro-1-pentanol (5H 4:1 FTOH); 6:2 fluorotelomer sulfonate (6:2 FTS); 6:2 chlorinated polyfluorinated ether sulfonate (F-53B); 8:2 fluorotelomer carboxylic acid (8:2 FTCA); ammonium 2,3,3,3-tetrafluoro-2-(heptafluoropropoxy)propanoate (GenX); bio-accumulation factor (BAF); bio-concentration factor (BCF); biomagnification factor (BMF); biota-sediment accumulation factor (BSAF); effective concentrations (EC); extracellular signal-regulated kinase (ERK); final length of the muzzle (SVL); heneicosafluoroundecanoic acid (PFUnDA); inhibitory concentration (IC); kinetic constant (k); lethal concentration (LC); liver fatty acid binding protein (LFABP); mass index of the scale (SMI); No observed effect concentration (NOEC (No Observed Effect Concentration); N-terminal c-Jun (JNK); Organization for Economic Cooperation and Development (OECD); perfluoroalkane sulphonic acids (PFSA); perfluoroalkanoic carboxylic acids (PFCA); perfluoroalkyl phosphonic acid (PFPAs); perfluoroalkylic acids (PFAA); perfluorobutanesulfonic acid (PFBS); perfluorobutanoic acid (PFBuA); perfluorobutyric acid (PFBA); perfluorodecane sulfonic acid (PFDS); perfluorodecanoic acid (PFDA); perfluorododecanoic acid (PFDoA); perfluorohexanoic acid (PFHxA); perfluoroheptanesulfonic acid (PFHpS); perfluoroheptanoic acid (PFHpA); perfluorohexanesulfonic acid (PFHxS); perfluorononanoic acid (PFNA) perfluorononanoic acid (PFNA); perfluorononenoxybenzenesulfonate (OBS); perfluorooctane sulfonamide (FOSA); perfluorooctanesulfonic acid (PFOS); perfluorooctanesulfonyl fluoride (POSF); perfluorooctanoic acid (PFOA); perfluoropentanoic acid (PFPeA); perfluoropolyethers (PFPeS); perfluoroundecanoic acid (PFUnA); persistent organic pollutants (POPs); physiologically based pharmacokinetic (PBPK); poly- and perfluoroalkyl substances (PFAS); poly- and perfluorinated compounds (PFCs); steady state bio-accumulation factor (BAF_SS_); steady state bio-concentration factor (BCF_SS_); trophic magnification factors (TMF).
